# The Medical Epitome Series—Obstetrics

**Published:** 1904-03

**Authors:** 


THE MEDICAL EPITOME SERIES—OBSTETRICS. A Manual for Students and Practitioners. By W. P. Manton, M. D. Series edited by V. C. Pedersen, A. M., M. D. Illustrated with 82 engravings. Published by Lea Brothers & Co., New York and Philadelphia.

This little volume, of course, contains only the essentials of its subject, but it presents them in such a concise, clear and definite manner that we feel no hesitancy in recommending it as a thoroughly scientific work. It will be found to be of much practical use to the student and the beginner in the subject.

